# The association between EKG abnormalities and the development of microalbuminuria in type 2 diabetes

**DOI:** 10.1097/MD.0000000000028018

**Published:** 2021-12-23

**Authors:** Yu-Kang Chang, Hueng-Chuen Fan, Chih-Cheng Hsu, Paik-Seong Lim

**Affiliations:** a Department of Medical Research, Tungs’ Taichung MetroHarbor Hospital, Taichung, Taiwan; b Department of Nursing, Jenteh Junior College of Medicine, Nursing and Management, Miaoli, Taiwan; c Department of Pediatrics, Tungs’ Taichung MetroHarbor Hospital, Taichung, Taiwan; d Department of Rehabilitation, Jenteh Junior College of Medicine, Nursing and Management, Miaoli, Taiwan; e Institute of Population Health Sciences, National Health Research Institutes, Miaoli, Taiwan; f Department of Health Services Administration, China Medical University, Taichung, Taiwan; g Department of Family Medicine, Min-Sheng General Hospital, Taoyuan, Taiwan; h Division of Renal Medicine, Tungs’ Taichung MetroHarbor Hospital, Taichung, Taiwan.

**Keywords:** diabetes mellitus, diabetic nephropathy, DMIDS, EKG, end-stage renal disease

## Abstract

Microalbuminuria is associated with both with chronic kidney disease and various cardiovascular abnormalities. Given the common use of electrocardiograms (EKGs) in diagnosing cardiovascular dysfunction, this study is analyzing the relationship between EKG abnormalities and diabetic nephropathy in type 2 diabetes mellitus (DM) patients. The enrollments of this study were from the 10-year follow-up data (2003–2012) of the Diabetes Management through an Integrated Delivery System project. All study subjects underwent at least 1 EKG measurement. The urinary microalbuminuria was recorded annually. The logistic regression model was used to evaluate the association between EKG abnormalities and the occurrence of diabetic nephropathy in type 2 DM patients. The total of 1189 patients with type 2 DM are included in this study and a total of 552 patients had microalbuminuria during a 10-year follow-up. A significantly higher odds ratio of microalbuminuria occurrence (4.85) was found in the patients with premature supraventricular contraction or tachycardia compared to those without EKG abnormalities. The odds ratios of microalbuminuria occurrence were 1.00, 2.43, 2.64, and 2.98, respectively, for patients with insulin resistance in the Q (quartile) 1(as the reference), Q2, Q3, and Q4, respectively. Our findings can serve as a reference for the association between EKG abnormalities and the development of microalbuminuria in type 2 diabetes.

## Abstract 2

Supplemental Digital Content is available in the text

## 1 Introduction

End-stage renal disease often leads to coronary artery disease, electrocardiogram (EKG) abnormalities (including QT interval prolongation and increased heart rate variability), atrial fibrillation, and ventricular arrhythmia, and even to sudden death.^[1–5]^ Microalbuminuria is often used as an indicator in the early stages of chronic kidney disease, as well as for evaluating vascular endothelial dysfunction in the early stages of cardiovascular disease,^[6,7]^ predicting the risks of myocardial infarction and stroke,^[8–10]^ and detecting left ventricular hypertrophy and abnormalities in left ventricular diastolic function and systolic function.^[11–13]^ Relatedly, chronic kidney disease and microalbuminuria are strongly associated with cardiovascular disease and the functions of the cardiac system, and in previous studies. Microalbuminuria has been used as an indicator of the prevalence and prognosis of hypertension.^[7,8]^ Evidence-based studies have also demonstrated the usefulness of microalbuminuria in the assessment of cardiovascular disease, as well as for effectively predicting the risk of myocardial infarction and stroke^[11–13]^ and evaluating vascular endothelial dysfunction in the early stages of cardiovascular disease.^[14,15]^ Moreover, microalbuminuria can be used to assess cardiac and vascular damage. Studies have proven that increased albuminuria levels are correlated positively with cardiac function abnormalities,^[16]^ and can even be used to assess left ventricular hypertrophy and abnormalities in left ventricular diastolic function and systolic function.^[10,17–19]^

In light of the association between microalbuminuria and cardiovascular abnormalities, as well as the common use of EKGs in the diagnosis of cardiovascular function abnormalities, there may be an association between EKG abnormalities and the occurrence of microalbuminuria. Recently, in the Italy-Developing Education and awareness on Microalbuminuria in patients with hypertensive Disease study, Sciarretta et al^[16]^ took the EKG and microalbuminuria measurements of 4121 hypertensive patients with no apparent cardiovascular disease. The results revealed that the odds ratios of acquiring microalbuminuria and acquiring chronic kidney disease were 1.81 and 1.66 times higher, respectively, for patients with EKG abnormalities than for those without EKG abnormalities. Furthermore, the odds ratios of microalbuminuria occurrence were 1.95, 1.84, and 1.87 higher for those with intraventricular conduction defects, ventricular repolarization alterations, and left-axis deviation, respectively, than for those without these complications. Hence, a significant association exists between EKG abnormalities and the occurrence of microalbuminuria. However, since the aforementioned study was a cross-sectional study, it did not clarify the causal relationship between EKG abnormalities and chronic kidney disease. Furthermore, to the best of our knowledge, there have been no studies on the relationship between EKG abnormalities and nephropathy in type 2 diabetes mellitus (DM) patients. As an EKG is a common diagnostic tool for cardiovascular function abnormalities, it is important to clarify the association between EKG conduction functions and pathological changes in diabetic nephropathy, so as to facilitate early diagnosis and treatment in clinical practice. Therefore, the objective of this study was to analyze the prevalence of various EKG abnormalities in type 2 diabetes mellitus patients in Taiwan by utilizing the long-term cohort data in the Diabetes Management through an Integrated Delivery System (DMIDS) project. Similarly, the relationship between EKG abnormalities in type 2 DM patients and diabetic nephropathy was also investigated in this study.

## 2 Methods

### 2.1 Study population

**Figure 1:**
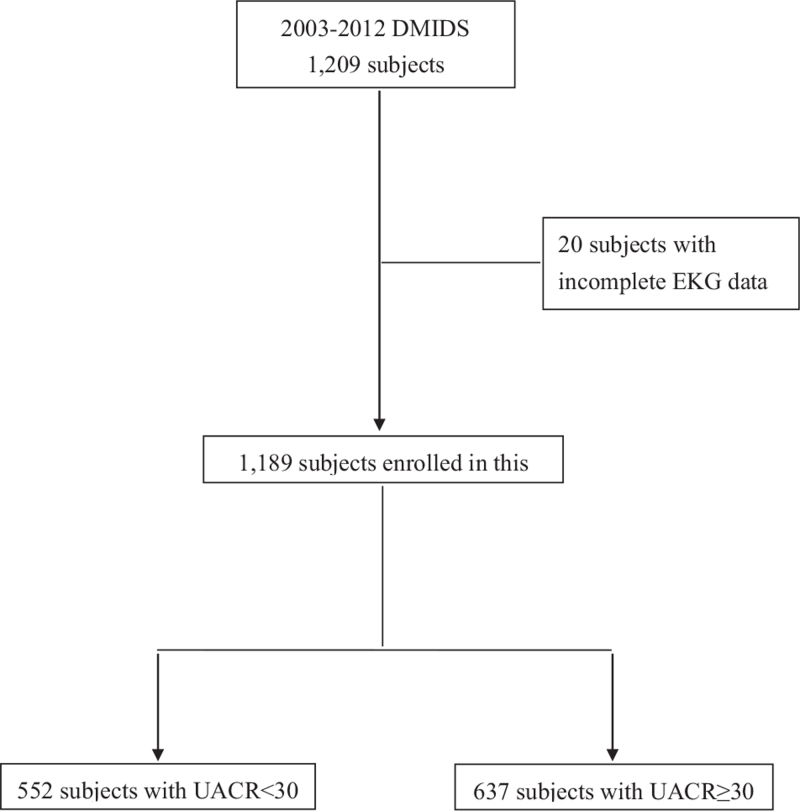
The flow chart of this study.

The data used in this study came from patients with type 2 DM who enrolled in the DMIDS project (ClinicalTrials.gov, NCT00288678), which was the first Taiwan randomized clinical trial aimed to evaluate the effectiveness via an integrated care program for diabetic patients. To conduct an 5-year intervention for the period from 2003 to 2007, the DMIDS study was transformed into a cohort study (from 2008 to 2012) so as to observe the related risk factors of diabetic nephropathy.^[20]^ The inclusion criteria of study patients were diagnosed with type 2 DM. Only if they met any exclusion criteria including being younger than 30 years old, older than 70 years old, being pregnant, or having major diabetic complications, such as a leg amputation, uremia, or hospitalization, in the previous year because of acute myocardial infarction, heart failure, or stroke.^[21]^ There were 36 local clinics chosen at northern, central, and southern in Taiwan, and the study patients were started from 2003 to 2007 and followed-up to the end of 2012. The flow chart of this study was presented in Figure 1. This study was approved by the institutional review board of the Tungs’ Taichung MetroHarbor Hospital in Taichung, Taiwan (107058).

### 2.2 Measurements

All of the patients who took part in the DMIDS long-term cohort project underwent at least 1 EKG measurement (the average number of measurements was 3). In the present study, the results of these EKG measurements were interpreted by a commissioned cardiologist according to the Minnesota Code,^[22]^ after which the interpreted results were collated and classified. Any EKG abnormalities and the type of each abnormality were determined according to the Minnesota Code classification system, with the EKG findings for each patient being classified as major, minor, or no abnormalities.

Major abnormalities included Q-RS wave abnormalities (codes 1-1 to 1-2-8), left ventricular hypertrophy (code 3-1), Wolff-Parkinson-White syndrome (a type of congenital cardiac arrhythmia; code 6-4-1 or 6-4-2), complete bundle branch block or intraventricular block (code 7-1-1, 7-2-1, 7-4, or 7-8), and atrial fibrillation or atrial flutter (code 8-3). Minor abnormalities included ST-T segment changes (codes 4-1, 4-2, 5-1, and 5-2).^[23]^ Then, differences in the baseline demographic variables (age, gender, education level, age of diabetes diagnosis, and smoking history) and antihypertensive, antidiabetic drug, and anti-hyperlipidemia use of the patients with major, minor, or no EKG abnormalities were compared. The comparisons of baseline biochemical indicators (body mass index, waist circumference, glomerular filtration rate, systolic pressure, diastolic pressure, glycated hemoglobin (Hb) level, total cholesterol level, triglyceride level, high-density lipoprotein (HDL) level, leukocyte count, fibrinogen level, high-sensitivity C-reactive protein level, and ferritin level), and antihypertensive and antidiabetic drug use of the patients with major, minor, or no EKG abnormalities were included in this study.

Patients with a urine albumin-to-creatinine ratio (UACR) of at least 30 mg/dL after 2 consecutive tests were deemed to have microalbuminuria. But those with a red blood cell, leukocyte, or epithelial cell count of 5 per high power field and above in the microscopic urinalysis or those with apparent bacterial responses in their urine were excluded. The logistic regression models were used to explore the differences in the odds ratios of microalbuminuria occurrence among the 3 groups of patients (that is, those with major, minor, or no EKG abnormalities).

Next, the patients’ EKG abnormalities were further classified as Table S1, Supplemental Digital Content, http://links.lww.com/MD2/A791. The differences in the baseline demographic variables, biochemical indicators, and antihypertensive and antidiabetic drug use of the patients with these different EKG abnormalities were then also compared with those of the patients without EKG abnormalities. Lastly, the odds ratios and the differences in microalbuminuria occurrence among the patients with and without different EKG abnormalities were explored in this study.

For each patient, the demographics, smoking history, age at diagnosis of diabetes (in years), waist circumference (cm), and blood pressure (mm Hg) were measured at recruitment. The triglyceride (mg/dL), HDL cholesterol (mg/dL), and HbA1c levels (%), as well as the UACR, were measured at recruitment and during the follow-up period. Blood and urine specimens collected from each participating clinic were stored in containers at 2 to 8°C and express transported to a central laboratory within 24 hours. All measurements were obtained and analyzed using the same protocols during the study period. UACR has a relatively high intraindividual variability.^[24,25]^ Therefore, average UACR values for each patient were used to minimize the effects of such variability and to better represent the general condition of the patient's albuminuria. Specifically, the entry UACR was calculated as the geometric mean of the first 2 UACRs, and the final UACR was calculated as the geometric mean of the last 2 UACRs. The entry and final times were the means of the calendar years when the first and last 2 UACRs, respectively, were obtained. The annual increase in UACR was calculated as [log (final UACR) − log (entry UACR)]/(follow-up years).

### 2.3 Statistical analyses

The dependent variable was the annual increase in UACR. The covariates were demographics (age at baseline entry time, sex, education, and smoking status), disease history (duration of diabetes at recruitment and age at diagnosis of diabetes), baseline measurements (waist circumference, blood pressure, triglyceride level, HDL cholesterol level, and HbA1C level), and climatic factors (ambient temperature and relative humidity).

The differences among the patients with major, minor, and no EKG abnormalities were compared in this study. In addition, the differences in the baseline demographic data (age, gender, education level, age of diabetes diagnosis, and smoking history), baseline biochemical indicators (waist circumference, body mass index, HbA1c, systolic pressure, diastolic pressure, Homeostatic Model Assessment for Insulin Resistance, total cholesterol level, non-HDL level, triglyceride level, HDL level, low-density lipoprotein level), and antihypertensive, antidiabetic and anti-hyperlipidemia drug use of the patients with different EKG abnormalities were also compared with those of the patients without EKG abnormalities. The differences among these variables were analyzed respectively through the *t*-test, analysis of variance (*f*-test), and chi-square test. Univariate and multivariate logistic regression analyses were also used to analyze the data of the patients with major, minor, and no EKG abnormalities, as well as the odds ratios of microalbuminuria occurrence in the patients with and without different EKG abnormalities. The differences in the variables were further compared.

All analyses were performed using JMP software (Version 9.4 SAS Institute Inc., Cary, NC), and a 2-sided *P* value < .05 was considered statistically significant.

## 3 Results

A total of 1209 patients were recruited in this study. After omitting 20 patients with incomplete EKG data, the remaining 1189 type 2 DM patients were analyzed. During the follow-up period that ended in 2012, a total of 552 patients had microalbuminuria. The flow chart of this study was shown in Figure 1. With regard to the distribution of different demographic variables and biochemical indicators, the patients with microalbuminuria had a longer average duration of type 2 DM compared to those without microalbuminuria (5.8 vs 4.4 years). They also had a higher glycated Hb level (8.8% vs 8.2%) and significantly higher systolic pressure, rate of hypertension, and usage rates of antihypertensive drugs such as angiotensin- converting enzyme inhibitors/angiotensin receptor blockers and calcium channel blocker. However, the usage rates of antidiabetic drugs such as sulfonylurea and biguanide in these patients were significantly lower. In terms of biochemical indicators, patients with microalbuminuria had a higher insulin resistance level (5 vs 3.9), total cholesterol level (197.2 vs 191.6 mmol/L), non-HDL level (149.2 vs 142.8 mg/dL), and triglyceride level (199.4 vs 162 mg/dL) than the patients without microalbuminuria, as shown in Table 1.

**Table 1:** Comparisons of the demographic and biochemical characteristics of the type 2 DM patients with and without microalbuminuria.

	Total	With microalbuminuria	Without microalbuminuria	
Variable	(n = 1189)	(n = 552)	(n = 637)	*P* value
Male, n (%)	570 (47.9)	262 (47.5)	308 (48.4)	.760
Education ≤6 yr, n (%)	676 (56.9)	326 (59.1)	226 (55.0)	.153
Mean age of diabetic occurrence, yr	55.9 ± 8.6	56.2 ± 8.5	55.7 ± 8.6	.360
Duration of diabetes, yr	5 ± 5.6	5.8 ± 6.1	4.4 ± 5.1	<.001
Smoking status, n (%)				.137
Nonsmoker	802 (67.5)	360 (65.3)	442 (69.4)	
Current smoker	386 (32.5)	191 (34.7)	195 (30.6)	
Waist circumference, cm	87.9 ± 10.4	88.3 ± 10.2	87.6 ± 10.6	.276
BMI, kg/m^2^	25.9 ± 3.8	26.2 ± 3.9	25.7 ± 3.7	.026
HbA1c, mmol/mol (mean ± SD, %)	8.5 ± 2.0	8.8 ± 2.0	8.2 ± 1.9	<.001
SBP, mm Hg	129.9 ± 16.5	131.7 ± 16.7	128.3 ± 16.2	<.001
DBP, mm Hg	81.9 ± 42.5	81.5 ± 9.5	82.2 ± 57.4	.736
Antihypertensive use, n (%)				
ACEi/ARB	538 (45.3)	285 (51.6)	253 (39.7)	<.001
CCB	459 (38.6)	238 (43.1)	221 (34.7)	.003
β-blocker	400 (33.6)	199 (36.1)	201 (31.6)	.102
Diuretic	251 (21.1)	124 (22.5)	127 (19.9)	.287
Antidiabetic use, n (%)				
Sulfonylurea	1087 (91.4)	516 (93.5)	571 (89.6)	.018
Biguanide	986 (82.9)	475 (86.1)	511 (80.2)	.008
Thiazolidinedione	137 (11.5)	66 (12.0)	71 (11.2)	.662
α-glucosidase inhibitor	72 (6.1)	36 (6.5)	36 (5.7)	.530
Meglitinide	34 (2.9)	20 (2.2)	14 (3.6)	.141
Anti-hyperlipidemia, n (%)				
Statin	44 (3.7)	20 (3.6)	24 (3.8)	.895
Hypertension (≥130/85 or on H/T drug), n (%)	654 (55.0)	349 (63.2)	305 (47.9)	<.001
Insulin resistance (HOMA-IR)	4.5 ± 7.5	5 ± 8.6	3.9 ± 6.2	.016
Total cholesterol, mg/dL	194.2 ± 43	197.2 ± 44.6	191.6 ± 41.5	.025
Non-HDL cholesterol, mg/dL	145.8 ± 41.8	149.2 ± 43.1	142.8 ± 40.5	.008
Triglycerides, mg/dL	179.4 ± 177.4	199.4 ± 214.3	162 ± 135.4	<.001
HDL cholesterol, mg/dL	48.3 ± 13	47.9 ± 12	48.7 ± 13.7	.303
LDL cholesterol, mg/dL	124.6 ± 36.3	125.7 ± 36.5	123.7 ± 36.1	.348

ACEI = angiotensin- converting enzyme inhibitors, ARB = angiotensin receptor blockers, BMI = body mass index, CCB = calcium channel blocker, DBP = diastolic blood pressure, DM = diabetes mellitus, HbA1c = hemoglobin A1c, HDL = high-density lipoprotein, HOMA-IR = Homeostatic Model Assessment for Insulin Resistance, LDL = low-density lipoprotein, SBP = systolic blood pressure, SD = standard deviation.

The patients were further classified according to their EKG abnormalities as those with major, minor, and no abnormalities. In terms of the distributions of different baseline demographic variables and biochemical indicators, the patients with major EKG abnormalities were mostly male, had a smoking history, had a higher rate of hypertension, higher usage rates of antihypertensive drugs such as converting enzyme inhibitors/angiotensin receptor blockers and calcium channel blocker, and higher usage rates of antidiabetic drugs. The mean age at diabetes diagnosis, total cholesterol level, non-HDL level, and low-density lipoprotein level were also significantly higher in the patients with major EKG abnormalities. On the other hand, the patients with minor EKG abnormalities mostly had lower education levels and were nonsmokers. They also had significantly higher usage rates of antidiabetic drugs such as β-blockers and antihypertensive drugs such as sulfonylurea and α-glucosidase inhibitors, as shown in Table 2.

**Table 2:** Comparisons of the demographic and biochemical characteristics of the type 2 diabetes patients with major, minor, and no EKG abnormalities.

Variable	Major EKG abnormalities	Minor EKG abnormalities	No EKG abnormalities	*P* value
Male, n (%)	117 (54.9)	31 (28.4)	422 (48.7)	<.001
Education ≤ 6 yr, n (%)	128 (60.1)	79 (72.5)	469 (54.1)	<.001
Mean age of diabetic occurrence, yr	58.7 ± 7.9	57.4 ± 8.3	55.1 ± 8.6	<.001
Duration of diabetes, yr	5.3 ± 5.9	5.4 ± 4.9	4.9 ± 5.6	.331
Smoking status, n (%)				.028
Nonsmoker	130 (61.0)	82 (75.2)	590 (68.1)	
Current smoker	83 (39.0)	27 (24.8)	276 (31.9)	
Waist circumference, cm	88.2 ± 9.8	87.9 ± 9.6	87.8 ± 10.7	.699
BMI (kg/m^2^)	25.8 ± 3.6	26.3 ± 3.7	25.9 ± 3.8	.876
HbA1c (%)	8.5 ± 2.1	8.7 ± 2.0	8.5 ± 1.9	.155
SBP (mm Hg)	132.7 ± 18	133.1 ± 18	128.8 ± 15.8	<.001
DBP (mm Hg)	81.2 ± 11.1	81.8 ± 10.2	82.1 ± 49.3	.775
Hypertension (≥130/85 or on HTN drug), n (%)	143 (67.1)	71 (65.1)	440 (50.8)	<.001
Antihypertension use, n (%)				
ACEi/ARB	121 (56.8)	58 (53.2)	359 (41.4)	<.001
CCB	106 (49.8)	47 (43.1)	306 (35.3)	<.001
β-blocker	78 (36.6)	51 (46.8)	271 (31.3)	.003
Diuretic	54 (25.4)	28 (25.7)	169 (19.5)	.081
Antidiabetic use, n (%)				
Sulfonylurea	186 (86.4)	106 (97.3)	797 (91.9)	.003
Biguanide	185 (86.9)	83 (76.2)	718 (82.8)	.053
Thiazolidinedione	24 (11.3)	13 (11.9)	100 (11.5)	.985
α-glucosidase inhibitor	9 (4.2)	14 (12.8)	49 (5.7)	.006
Meglitinide	9 (4.2)	2 (1.8)	23 (2.7)	.372
Anti-hyperlipidemia, n (%)				
Statin	2 (5.6)	2 (2.8)	3 (3.3)	.032
Insulin resistance (HOMA-IR)	3.6 ± 3.9	6.3 ± 14.1	4.4 ± 7	.521
Total cholesterol (mg/dL)	199.6 ± 45.2	197.9 ± 39.8	192.4 ± 42.8	.018
Non-HDL cholesterol (mg/dL)	150.3 ± 42.4	148.4 ± 39.1	144.4 ± 41.9	.048
Triglycerides (mg/dL)	170.7 ± 163.3	186.3 ± 165.3	180.7 ± 182.3	.535
HDL cholesterol (mg/dL)	49.3 ± 13.8	49.5 ± 12.7	48 ± 12.8	.134
LDL cholesterol (mg/dL)	129.8 ± 38.9	126.5 ± 35.1	123.2 ± 35.7	.014

HOMA-IR = Insulin (μU/mL) × Blood sugar (mmol/L)/22.5. Major EKG abnormalities: Q-RS wave abnormalities (codes 1-1 to 1-2-8), left ventricular hypertrophy (code 3-1), Wolff-Parkinson-White syndrome (a type of congenital cardiac arrhythmia; code 6-4-1 or 6-4-2), complete bundle branch block or intraventricular block (code 7-1-1, 7-2-1, 7-4, or 7-8), and atrial fibrillation or atrial flutter (code 8-3). Minor EKG abnormalities: ST-T segment changes (codes 4-1, 4-2, 5-1, and 5-2). ACEI = angiotensin- converting enzyme inhibitors, ARB = angiotensin receptor blockers, BMI = body mass index, CCB = calcium channel blocker, DBP = diastolic blood pressure, EKG = electrocardiogram, HbA1c = hemoglobin A1c, HDL = high-density lipoprotein, HTN = antihypertensive, HOMA-IR = Homeostatic Model Assessment for Insulin Resistance, LDL = low-density lipoprotein, SBP = systolic blood pressure.

With regard to the relationship between EKG abnormalities and the incidence of microalbuminuria, after adjusting for the basic demographic variables and relevant biochemical indicators, the odds ratios of microalbuminuria occurrence for the patients with major EKG abnormalities and the patients with minor EKG abnormalities were not statistically significant. However, a significantly higher odds ratio of microalbuminuria occurrence (4.85) was observed in the patients with premature supraventricular contraction or tachycardia compared to those without these conditions.

In terms of other biochemical indicators, the odds ratio of microalbuminuria occurrence was 1.55 in patients with a glycated hemoglobin level of 9% or above compared to those with a level below 7%; 1.51 in patients with a blood pressure of 140/90 mm Hg or above or those using antihypertensive drugs compared to those with a blood pressure below 120/80 mm Hg; and 4.75 in patients with an ACR of 10 mg/g or above compared with those whose ACR was less than 10 mg/g. Furthermore, the odds ratios of microalbuminuria occurrence were 2.43, 2.64, and 2.98, respectively, for patients with insulin resistance in the Q2, Q3, and Q4 quartiles compared to those in the Q1 quartile. These findings are summarized in Table 3.

**Table 3:** Risk factors associated with the development of microalbuminuria among subjects with type 2 diabetes.

	Crude OR (odds ratio)	*P* value	Multiple OR (odds ratio)	*P* value
Male, n (%)	0.97 (0.77∼1.21)	.760	1.07 (0.75–1.51)	.718
Duration of Diabetes, yr	1.18 (0.94–1.49)	.153	1.17 (0.87–1.58)	.306
Mean age of diabetic occurrence, yr (per add 1 yr)	0.99 (0.97–1.00)	.058	0.99 (0.97–1)	.096
Duration of diabetes (>5 yr)	1.58 (1.24–2.02)	<.001	1.31 (0.97–1.78)	.080
Smoker/nonsmoker	1.20 (0.94–1.53)	.137	1.13 (0.8–1.62)	.489
Waist circumference (>90/80 cm)	1.24 (0.99–1.56)	.067	1.02 (0.77–1.35)	.893
HbA1c 7–9/<7 (%)	1.31 (0.97–1.76)	.080	1.21 (0.86–1.69)	.284
HbA1c ≥9/<7 (%)	2.17 (1.60–2.995)	<.001	1.55 (1.08–2.23)	.018
LDL >100 mg/dL or anti-hyperlipidemia use	1.04 (0.79–1.36)	.793	0.89 (0.66–1.21)	.464
BP 120–139/80–89/BP <120/80 (mm Hg)	1.87 (1.48–2.36)	<.001	1.51 (1.07–2.13)	.019
BP ≥140/90 or antihypertension use/BP <120/80 (mm Hg)	2.03 (1.55–2.66)	<.001	1.32 (0.89–1.96)	.170
Insulin resistance (HOMA-IR)				
Quartile 2 (1.4–2.9)/quartile 1 (<1.4)	1.73 (1.26–2.36)	<.001	2.43 (1.7–3.49)	<.001
Quartile 3 (3–4.9)/quartile 1 (<1.4)	2.13 (1.54–2.39)	<.001	2.64 (1.84–3.79)	<.001
Quartile 4 (≥5)/quartile 1 (<1.4)	2.81 (2.03–3.88)	<.001	2.98 (2.07–4.3)	<.001
UACR ≥10 (mg/g)	4.99 (3.87–6.44)	<.001	4.75 (3.61–6.25)	<.001
EKG				
Main abnormal	1.25 (0.92–1.68)	.151	4.75 (3.61–6.25)	.404
Second abnormal	1.35 (0.91–2.02)	.137	1.16 (0.82–1.63)	.644
Supraventricular premature beats or tachycardia	7.33 (5.56–9.67)	<.001	4.85 (3.55–6.62)	<.001
Atrial fibrillation	1.45 (0.39–5.42)	.583	1.38 (0.33–5.74)	.662
sinus tachycardia	1.32 (0.65–2.67)	.441	0.93 (0.43–2.01)	.844
Left bundle branch block	1.16 (0.16–8.23)	.886	1.31 (0.152–11.37)	.804
Right bundle-branch block	0.86 (0.44–1.70)	.668	0.68 (0.32–1.45)	.321
Repolarization abnormalities	1.52 (1.15–2.01)	.004	1.27 (0.93–1.75)	.135
Myocardial necrosis	1.15 (0.79–1.68)	.466	1.08 (0.70–1.67)	.719
Left-axis deviation	0.99 (0.62–1.57)	.960	0.95 (0.57–1.59)	.836

EKG = electrocardiogram, HbA1c = hemoglobin A1c, HOMA-IR = Homeostatic Model Assessment for Insulin Resistance, LDL = low-density lipoprotein, OR = odds ratio, UACR = urine albumin-to-creatinine ratio.

## 4 Discussion

With respect to the relationship between type 2 DM patients with EKG abnormalities and their acquisition of diabetic nephropathy, the results of this study indicated no significant correlations between the incidence of microalbuminuria and either major or minor EKG abnormalities. However, the results did indicate a significant correlation between the occurrence of microalbuminuria and the EKG abnormalities of premature ventricular contractions and tachycardia, as the odds ratio of having microalbuminuria among patients with those abnormalities was 4.85 compared to those without these EKG abnormalities. The risk factors for having microalbuminuria were identified as having poor blood sugar control, hypertension, and an ACR of 10 mg/g and above. Previous studies have shown that microalbuminuria is not only a predictive factor of cardiovascular disease,^[26,27]^ but that it also is significantly correlated with EKG abnormalities.^[27,28]^

In a study by Diercks et al,^[29]^ 7579 nondiabetic patients aged 20 to 74 years old were analyzed. The results revealed a significant correlation between the occurrence of microalbuminuria and ischemic EKG abnormalities, with the odds ratios for microalbuminuria occurrence being 1.61 and 1.43, respectively, in patients with major and minor ischemic EKG abnormalities. In another study, Sciarretta et al^[16]^ investigated the correlation between EKG abnormalities and microalbuminuria occurrence in 4121 hypertensive patients. The results suggested that there is a significant correlation between EKG abnormalities and microalbuminuria occurrence, with an overall odds ratio of 1.81. More specifically, the EKG abnormalities of intraventricular conduction blocks, ventricular repolarization variations, and left axis deviation were positively correlated with the incidence of microalbuminuria, with odds ratios of 1.95, 1.84, and 1.87, respectively, for these 3 conditions.

In the present study, however, statistically significant correlations between EKG abnormalities and microalbuminuria occurrence were observed only in patients with the abnormalities of premature supraventricular contractions and tachycardia, whereas no statistically significant correlations were found in patients with other EKG abnormalities. This could be attributable to the fact that the type 2 DM patients included in this study already had a high risk of microalbuminuria, such that microalbuminuria could have occurred in many of them even before they had EKG abnormalities. On the other hand, the research population of this study is ethnically different from the European^[16,29]^ and South African^[28]^ in precious studies.

Furthermore, the EKG abnormalities caused by cardiovascular disease might take a long time to occur, such that the period of time spent tracking EKG results in this study might not have been long enough for the observed trends to develop into statistically significant correlations. Moreover, due to the insufficient observation period and the lack of a long-term follow-up in its research design, this study could not deduce any causal relationships between EKG abnormalities and microalbuminuria occurrence. In order to overcome these limitations, a longer follow-up period and a cohort study design could be employed in future studies. These approaches can facilitate the clarification of the causal relationships, if any, between EKG abnormalities in type 2 DM patients and the occurrence of microalbuminuria.

While previous studies have reported significant correlations between the occurrence of microalbuminuria and EKG abnormalities, the present study found statistically significant correlations between EKG abnormalities and microalbuminuria occurrence only in patients with the abnormalities of premature supraventricular contractions and tachycardia, while no statistically significant correlations were found in patients with other EKG abnormalities. However, the lack of more conclusive findings may have been attributable to the characteristics of the investigated patients, as well as the insufficient observation period and lack of a long-term follow-up in the design of the current study. In order to overcome these limitations, a longer follow-up period and a cohort study design could be employed in future studies. These approaches can facilitate the clarification of the causal relationships, if any, between EKG abnormalities in type 2 DM patients and the occurrence of microalbuminuria.

## Author contributions

**Conceptualization:** Paik Seong Lim.

**Data curation:** Yu-Kang Chang, Hueng-Chuen Fan, Chih-Cheng Hsu.

**Formal analysis:** Yu-Kang Chang.

**Investigation:** Yu-Kang Chang.

**Methodology:** Yu-Kang Chang.

**Project administration:** Yu-Kang Chang.

**Resources:** Chih-Cheng Hsu.

**Supervision:** Paik Seong Lim.

**Visualization:** Paik Seong Lim, Chih-Cheng Hsu.

**Writing – original draft:** Paik Seong Lim, Hueng-Chuen Fan.

**Writing – review & editing:** Paik Seong Lim, Chih-Cheng Hsu.
